# Role of Glutathione in Alleviating Chilling Injury in Bovine Blastocysts: Mitochondrial Restoration and Apoptosis Inhibition

**DOI:** 10.3390/antiox15010148

**Published:** 2026-01-22

**Authors:** Jingyu Ren, Fuhan Liu, Gang Liu, Biao Wang, Jie Zhu, Yongbin Liu, Yanfeng Dai

**Affiliations:** 1 Key Laboratory of Herbivorous Livestock Reproductive Regulation, National Sheep Genetic Evaluation Center, Inner Mongolia University, Hohhot 010030, China; 31608050@mail.imu.edu.cn (J.R.);; 2 Beijing Jingwa Agricultural Science & Technology Innovation Center, Beijing 101205, China; 3 Clinical Medicine Research Center, Affiliated Hospital of Inner Mongolia Medical University, 1 Tongdao North Street, Hohhot 010050, China; 4 Animal Husbandry Institute, Inner Mongolia Academy of Agricultural & Animal Husbandry Sciences, No. 22 Zhaowuda Road, Hohhot 010031, China; 5 Department of Animal Genetics, Breeding and Reproduction, College of Animal Science, Inner Mongolia Agricultural University, Hohhot 010070, China

**Keywords:** glutathione (GSH), bovine blastocyst, 4 °C preservation, oxidative stress, mitochondrial function, apoptosis, embryonic developmental potential

## Abstract

Short-term hypothermic storage at 4 °C represents a promising non-freezing alternative for transporting bovine embryos and synchronizing assisted reproductive procedures. However, chilling induces oxidative stress, mitochondrial dysfunction, and apoptosis, which markedly impair post-preservation embryonic viability. Glutathione (GSH), a key intracellular antioxidant, may mitigate these damaging effects, yet its protective mechanisms during bovine blastocyst hypothermic preservation remain unclear. Here, we investigated the impact of exogenous GSH supplementation on the survival, hatching ability, cellular integrity, mitochondrial function, and developmental potential of bovine blastocysts preserved at 4 °C for seven days. Optimization experiments revealed that 4 mM GSH provided the highest post-chilling survival and hatching rates. Using DCFH-DA, TUNEL, and γ-H2AX staining, we demonstrated that 4 °C preservation significantly increased intracellular reactive oxygen species (ROS), DNA fragmentation, and apoptosis. GSH supplementation markedly alleviated oxidative injury, reduced apoptotic cell ratio, and decreased DNA double-strand breaks. MitoTracker and JC-1 staining indicated severe chilling-induced mitochondrial suppression, including decreased mitochondrial activity and membrane potential (ΔΨm), which were largely restored by GSH. Gene expression analyses further revealed that chilling downregulated antioxidant genes (SOD2, GPX1, TFAM, NRF2), pluripotency markers (POU5F1, NANOG), and IFNT, while upregulating apoptotic genes (BAX, CASP3). GSH effectively reversed these alterations and normalized the BAX/BCL2 ratio. Moreover, SOX2/CDX2 immunostaining, total cell number, and ICM/TE ratio confirmed improved embryonic structural integrity and developmental competence. Collectively, our findings demonstrate that exogenous GSH protects bovine blastocysts from chilling injury by suppressing ROS accumulation, stabilizing mitochondrial function, reducing apoptosis, and restoring developmental potential. This study provides a mechanistic foundation for improving 4 °C embryo storage strategies in bovine reproductive biotechnology.

## 1. Introduction

Efficient preservation of in vitro-produced (IVP) bovine embryos is fundamental to the development of modern reproductive biotechnology, enabling embryo transport, genetic resource conservation, international germplasm exchange, and flexibility in synchronizing embryo transfer programs [1,2]. Conventional cryopreservation strategies—particularly slow freezing and vitrification—have greatly advanced commercial embryo transfer; however, these cryogenic methods still suffer from substantial limitations, including ice crystal formation, osmotic stress, zona pellucida hardening, and cellular damage associated with freeze–thaw cycles [3,4,5]. As a result, maintaining high post-thaw viability and developmental competence remains challenging, especially for IVP embryos, which are inherently more sensitive to cryo-injury than in vivo-derived embryos.

Short-term hypothermic preservation at 4 °C has emerged as a promising alternative to freezing-based approaches. Unlike cryopreservation, cooling embryos to near-physiological hypothermic temperatures slows cellular metabolism without causing intracellular ice formation, thereby providing a low-cost, equipment-free, and field-friendly method for temporary embryo preservation [6,7]. This strategy has been successfully applied in several mammalian species, including bovine, ovine, porcine, and murine models, and offers considerable logistical advantages in animal breeding industries [1,8,9]. Unlike conventional hypothermic storage, which is typically limited to 2–3 days for immediate transport [10], the present study targets a 7-day (168 h) preservation window. This duration is widely regarded as ‘medium-term’ or ‘prolonged’ liquid storage [6], filling the critical logistical gap between transient transport and indefinite cryopreservation. Establishing a one-week storage capacity is of paramount importance for the cattle industry, as it aligns seamlessly with standard weekly estrus synchronization and embryo transfer schedules. Furthermore, this window is sufficient to facilitate long-distance or even international transport of genetic resources without dependence on liquid nitrogen infrastructure.

However, despite its practicality, 4 °C preservation exposes embryos to sustained chilling stress, oxidative imbalance, and reductions in mitochondrial efficiency, leading to compromised embryo viability after rewarming and culture [11,12,13,14]. These chilling-induced damages remain a major barrier restricting broader application of 4 °C embryo storage. Oxidative stress is widely recognized as a central mediator of chilling injury. During hypothermic conditions, mitochondrial electron transport becomes inefficient, resulting in leakage of electrons and the excessive generation of ROS [15,16]. Accumulated ROS causes lipid peroxidation, protein oxidation, and DNA damage, triggering apoptosis and impairing cellular homeostasis [17]. Previous studies have demonstrated that cold-induced oxidative stress disrupts spindle organization, compromises membrane integrity, reduces ATP production, and accelerates embryonic arrest [5,18]. DNA double-strand breaks, marked by γ-H2AX accumulation, and apoptosis-related signaling pathways, including activation of BAX and caspase-3 (CASP3), are key downstream events that limit the developmental competence of chilled embryos [19,20]. Therefore, enhancing ROS detoxification and stabilizing mitochondrial function are essential strategies for improving hypothermic embryo preservation.

Glutathione (GSH), the most abundant intracellular antioxidant, plays vital roles in maintaining redox balance, detoxifying ROS, regulating mitochondrial activity, and protecting cellular structural integrity [21,22]. In oocytes and early embryos, GSH participates in sperm chromatin decondensation, pronuclear formation, epigenetic regulation, and embryonic genome activation [23,24]. Numerous studies have shown that endogenous GSH levels strongly correlate with oocyte developmental competence and embryo quality [25,26,27]. Supplementation of exogenous GSH has been widely employed to protect gametes or embryos from oxidative injury during in vitro maturation, fertilization, and cryopreservation. For example, in ovine and porcine oocytes, GSH improved post-warming survival by reducing ROS accumulation and stabilizing mitochondrial membrane potential [17,23]. Similarly, in vitrified bovine embryos, GSH alleviated cryo-induced apoptosis and enhanced blastocyst formation rates [4]. These findings suggest that GSH may exert broad cytoprotective effects during embryo preservation under stressful conditions.

Despite this knowledge, the role and molecular mechanisms of GSH in protecting bovine blastocysts during prolonged 4 °C storage remain largely unexplored. Compared with cryopreservation, hypothermic preservation exposes embryos to milder yet long-lasting oxidative challenges, and the biological responses to chilling may differ substantially from freezing-induced damage. To date, no comprehensive study has investigated how exogenous GSH modulates oxidative stress, mitochondrial function, apoptosis, DNA integrity, and developmental competence in bovine blastocysts subjected to extended 4 °C preservation.

The present study aimed to address these gaps by determining the optimal GSH concentration for maintaining embryo survival and hatching rates during storage at 4 °C, evaluating whether GSH alleviates chilling-induced oxidative damage such as ROS accumulation and DNA fragmentation, and assessing its effects on mitochondrial activity and membrane potential as key indicators of metabolic function. In addition, we investigated the regulatory influence of GSH on the expression of apoptosis-related and antioxidant genes, and examined whether GSH restores cellular integrity and developmental potential based on SOX2/CDX2 expression patterns, total cell number, and the ICM/TE ratio.

By integrating phenotypic analysis, molecular assays, mitochondrial indicators, and gene expression profiling, this study provides a comprehensive mechanistic understanding of how GSH ameliorates chilling injury in bovine blastocysts. Our findings highlight exogenous GSH as an effective and practical supplement for improving 4 °C embryo preservation, offering a promising non-freezing technology for assisted reproduction and genetic conservation in cattle. Moreover, the elucidated mechanisms provide insight into the oxidative stress–mitochondrial dysfunction–apoptosis axis that underlies chilling-mediated embryonic impairment, contributing to broader optimization of hypothermic preservation strategies in mammalian reproduction.

## 2. Materials and Methods

### 2.1. Ethics Statement

All procedures involving animals were conducted in accordance with the guidelines for the care and use of laboratory animals approved by the Institutional Animal Care and Use Committee of Inner Mongolia University. Ovaries were collected at a local abattoir from animals slaughtered for commercial meat production; therefore, no additional animal sacrifice was required for this study.

### 2.2. Collection of Ovaries and In Vitro Embryo Production

Bovine ovaries were obtained from a local slaughterhouse and transported to the laboratory within 2 h in sterile saline supplemented with 100 IU/mL penicillin and 100 μg/mL streptomycin (P/S, 15140122, Thermo Fisher, Beijing, China) at 30–35 °C to ensure oocyte competence and reduce pre-experimental stress variables. Cumulus–oocyte complexes (COCs) were aspirated from 2 to 8 mm follicles using an 18-gauge needle and selected based on uniform cytoplasm and compact cumulus layers. In vitro maturation (IVM), fertilization (IVF), and culture (IVC) procedures were performed following standard protocols.

Briefly, COCs were matured for 22–24 h in TCM-199 (11150067, Thermo Fisher, Shanghai, China) supplemented with 10% fetal bovine serum (FBS) (30044333, Thermo Fisher, Shanghai, China), 1 μg/mL estradiol (E8875, Sigma Aldrich, Shanghai, China), 0.5 μg/mL FSH (Sansheng, Ningbo, China), and 0.1 mM cysteine (M9768, Sigma Aldrich, Shanghai, China) at 38.5 °C in 5% CO_2_. Matured oocytes were inseminated with frozen–thawed bull semen at a concentration of 1 × 10^6^ sperm/mL and co-incubated for 6 h. Presumptive zygotes were cultured in SOF medium supplemented with 0.8% BSA (A3803, Sigma Aldrich, Shanghai, China) under mineral oil at 38.5 °C with 5% CO_2_, 5% O_2_, and 90% N_2_ until Day 6 (E6 blastocyst stage), when embryos of similar morphology were randomly assigned to experimental treatments. All experiments were performed in at least three independent biological replicates (three independent IVF sessions), with approximately 20–30 blastocysts allocated to each treatment group per replicate.

### 2.3. Preparation of Preservation Solutions and GSH Supplementation

The base medium for hypothermic preservation consisted of TCM-199 supplemented with 50% FBS and 25 mM HEPES (15630, Thermo Fisher, Shanghai, China). Glutathione (GSH; reduced form Sigma-Aldrich) was dissolved freshly in the preservation medium. Four treatment groups were prepared to determine the optimal antioxidant concentration: 0 mM GSH (Control), 2 mM GSH, 4 mM GSH, and 8 mM GSH. Embryos were preserved in 1.5 mL sterile tubes containing 500 μL of the assigned preservation solution. All procedures were performed under sterile conditions.

### 2.4. Hypothermic (4 °C) Preservation of Bovine Blastocysts

Day 6 blastocysts were washed twice in the designated preservation medium and transferred into tubes containing GSH-supplemented or control solutions. A HEPES-buffered medium (TCM-199 with 25 mM HEPES) was used specifically to maintain pH stability in ambient air conditions outside the incubator. The tubes were tightly sealed to minimize gas exchange and contamination and stored at 4 °C for 168 h (7 days). Storage temperature was continuously monitored using a digital thermometer (accuracy ± 0.1 °C) twice daily, and the tubes were kept static (upright) to prevent mechanical shear stress. At the end of the preservation period, embryos were passively rewarmed by holding the tubes at a controlled room temperature of approximately 25 °C for 20–30 min. To minimize osmotic shock, embryos were maintained in the preservation medium during warming and were transferred into fresh SOF medium only after the rewarming phase for re-culture at 38.5 °C.

Survival and hatching status were evaluated at 24 and 48 h post-warming. Embryos maintaining intact morphology with a visible blastocoel were considered surviving, whereas those exhibiting expanded blastocoels and/or complete escape from the zona pellucida were classified as hatched or hatching.

### 2.5. Detection of Intracellular ROS Levels (DCFH-DA Staining)

ROS levels were assessed using the DCFH-DA (S0033, Beyotime, Shanghai, China) fluorescent probe to detect general oxidative stress activity. Fresh, 4 °C-preserved, and 4 °C + GSH blastocysts were washed in PBS (14190250, Thermo Fisher, Beijing, China) containing 0.1% BSA and incubated with 10 μM DCFH-DA at 38.5 °C for 30 min in the dark. After washing, embryos were mounted on slides and imaged using a confocal laser scanning microscope (A1R, Nikon, Tokyo, Japan). Fluorescence intensity was quantified using ImageJ, version 1.54p, with a minimum of 30 embryos analyzed per group from three biological replicates.

### 2.6. TUNEL Assay for Detection of Apoptotic Cells

Apoptotic DNA fragmentation was detected using a commercial TUNEL (C1091, Beyotime, Shanghai, China) assay kit following the manufacturer’s protocol. Embryos were fixed in 4% paraformaldehyde (P1110, Solarbio, Beijing, China) for 30 min, permeabilized in 0.5% Triton X-100 (T8200, Solarbio, Beijing, China), and incubated with TUNEL reaction mixture for 1 h at 37 °C in the dark. Nuclei were counterstained with DAPI. TUNEL-positive cells were quantified and expressed as the apoptotic cell ratio. Fluorescence intensity was quantified using ImageJ, with a minimum of 30 embryos analyzed per group from three biological replicates.

### 2.7. Immunofluorescence Detection of γ-H2AX

DNA double-strand breaks were assessed using anti-γ-H2AX (ab81299, Abcam, Shanghai, China) antibody staining. Fixed and permeabilized blastocysts were blocked with 1% BSA and incubated overnight at 4 °C with primary antibody (anti-γ-H2AX, 1:500). After washing, embryos were incubated with Alexa Fluor-conjugated secondary antibody (with 1:300 dilutions in DPBS, ab150074, Abcam, Shanghai, China) for 1 h. Images were captured by confocal microscopy, and fluorescence intensity was quantified. Fluorescence intensity was quantified using ImageJ, with a minimum of 30 embryos analyzed per group from three biological replicates.

### 2.8. Mitochondrial Activity Assessment

Mitochondrial activity was evaluated using MitoTracker Red CMXRos (C1049, Beyotime, Shanghai, China). Embryos were incubated with 500 nM MitoTracker Red for 30 min at 38.5 °C, washed, and immediately imaged. The mean fluorescence intensity was calculated to reflect mitochondrial metabolic activity. Fluorescence intensity was quantified using ImageJ, with a minimum of 30 embryos analyzed per group from three biological replicates.

### 2.9. Assessment of Mitochondrial Membrane Potential

Mitochondrial membrane potential (ΔΨm) was assessed using JC-1 (C1049, Beyotime, Shanghai, China) dye. After incubation with 2 μM JC-1 for 30 min, embryos were washed and analyzed for J-aggregate (red) and J-monomer (green) fluorescence. The J-aggregate/J-monomer ratio was used to indicate ΔΨm. Fluorescence intensity was quantified using ImageJ, with a minimum of 30 embryos analyzed per group from three biological replicates.

### 2.10. Quantitative Real-Time PCR Analysis of Gene Expression

Total RNA was extracted from three independent biological replicates, each consisting of a pool of 8–10 blastocysts, using a PicoPure RNA isolation kit (KIT0204, Thermo Fisher Scientific, Waltham, MA, USA) cDNA synthesis was performed using a high-capacity reverse transcription kit (4368814, Thermo Fisher Scientific, Waltham, MA, USA). qPCR was conducted using SYBR Green chemistry (A25742, Thermo Fisher Scientific, Waltham, MA, USA) in a 20-μL reaction volume. Target genes included: antioxidant genes: SOD2, GPX1, TFAM, and NRF2; pro-/anti-apoptotic genes: BAX, BCL2, and CASP3; and developmental competence genes: POU5F1, NANOG, CDX2, and IFNT. β-actin was used as the internal control based on its reported stability in bovine embryos under stress conditions [28], and relative expression levels were calculated using the 2^−ΔΔCt^ method.

### 2.11. SOX2/CDX2 Immunofluorescence and Cell Allocation Analysis

To evaluate ICM and TE cell distribution, embryos were fixed, permeabilized, and blocked as described previously. Subsequently, they were incubated with anti-SOX2 (ab137385, Abcam, Shanghai, China) and anti-CDX2 (ab76541, Abcam, Shanghai, China) primary antibodies, followed by fluorescent secondary antibodies. Nuclei were counterstained with DAPI (C0065, Solarbio, Beijing, China). Confocal images were used to quantify total cell number, inner cell mass/trophectoderm (ICM/TE) ratio, and spatial localization of SOX2^+^ and CDX2^+^ cells, with a minimum of 30 embryos analyzed per group from three biological replicates.

### 2.12. Statistical Analysis

Data analysis was performed using SPSS 25.0 (IBM Corp., Armonk, NY, USA). The data were tested for normality using the Shapiro–Wilk test and for homogeneity of variance using Levene’s test. No outliers were excluded from the analysis. Differences among groups were evaluated using one-way ANOVA followed by Tukey’s post hoc test. All data are expressed as mean ± SEM. A *p*-value < 0.05 was considered statistically significant.

## 3. Results

### 3.1. Optimization of GSH Concentration for Improving Bovine Blastocyst Survival During 4 °C Preservation

**Figure 1:**
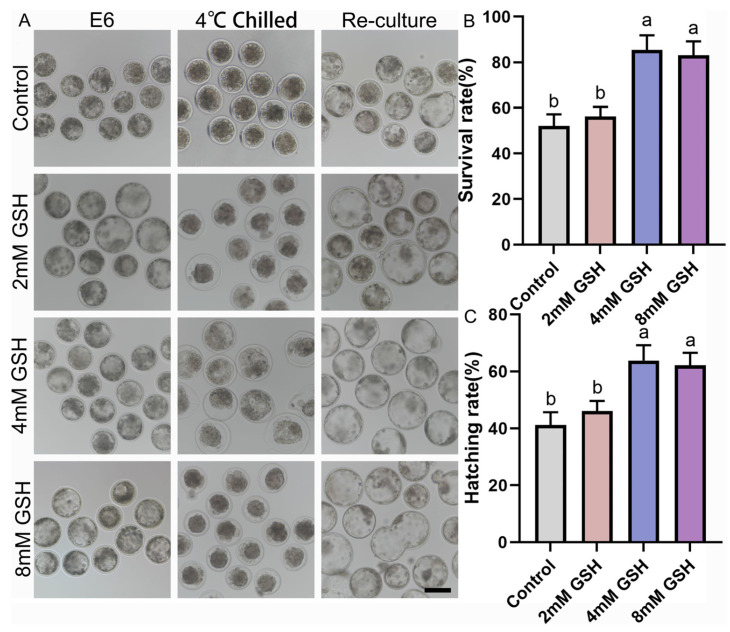
Effect of various concentrations of GSH supplementation on the survival and hatching rates of bovine blastocysts following 4 °C chilling; (**A**) Representative images of bovine blastocysts during 4 °C chilling and subsequent re-culture. Control: Blastocysts preserved in chilling solution without GSH supplementation. 2 mM GSH, 4 mM GSH, 8 mM GSH: Blastocysts preserved in chilling solution supplemented with 2 mM, 4 mM, and 8 mM GSH, respectively. E6: Blastocyst image before chilling. 4 °C Chilled: Blastocysts immediately after 168 h of 4 °C preservation. Re-culture: Blastocysts after re-culture following 4 °C preservation. Scale bar = 50 μm. (**B**) Statistical analysis of the survival rate of blastocysts after 4 °C chilling and re-culture. (**C**) Statistical analysis of the hatching rate of blastocysts after 4 °C chilling and re-culture. Note: Different lowercase letters in the bar charts indicate significant differences between groups (*p* < 0.05).

To determine the optimal antioxidant concentration for hypothermic storage, Day 6 bovine blastocysts were preserved at 4 °C for 168 h in medium supplemented with 0, 2, 4, or 8 mM GSH and subsequently re-cultured under standard conditions. Representative morphological changes during chilling and post-warming culture are shown in Figure 1A. Embryos in the control group (0 mM GSH) exhibited severe shrinkage, darker cytoplasm, and compromised blastocoel re-expansion after warming. In contrast, embryos preserved with 4 mM GSH maintained clearer blastocoel structures and greater morphological integrity. Consistent with these observations, quantitative analysis revealed that 4 mM GSH yielded the highest post-preservation survival and hatching rates (Figure 1B,C). Specifically, the survival rate of the 4 mM GSH group (85.39 ± 3.75%, n = 69) was significantly higher than that of the control group (51.96 ± 3.06%, n = 66; *p* < 0.05). Embryos preserved with 2 mM GSH (56.17 ± 2.45%, n = 62) displayed a slight improvement over controls but did not reach statistical significance. Embryos stored in 8 mM GSH (83.03 ± 3.49%, n = 58) also showed increased survival, but the value remained significantly lower than that of the 4 mM group (*p* < 0.05).

A similar pattern was observed for the hatching rate, where 4 mM GSH (63.85 ± 3.18%, n = 69) produced the most favorable outcomes, significantly surpassing both the control (41.16 ± 2.66%, n = 66; *p* < 0.05) and 2 mM groups (46.10 ± 2.15%, n = 62; *p* < 0.05). The 8 mM treatment (62.19 ± 2.52%, n = 58) significantly improved hatching compared with control embryos (*p* > 0.05), but showed no difference from the 4 mM group (*p* < 0.05). Collectively, these findings indicate that 4 mM GSH is the optimal concentration for protecting bovine blastocysts during prolonged 4 °C preservation, and this concentration was used in all subsequent experiments.

### 3.2. GSH Attenuates Chilling-Induced Oxidative Stress in Bovine Blastocysts

**Figure 2:**
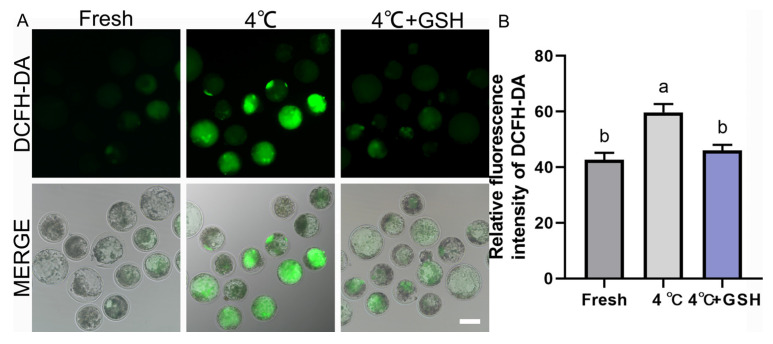
GSH alleviates 4 °C chilling-induced oxidative stress in bovine blastocysts; (**A**) Representative images of ROS levels detected by DCFH-DA fluorescence probe. Fresh: Non-chilled control blastocysts. 4 °C: Chilled control blastocysts. 4 °C + GSH: Blastocysts preserved with 4 mM GSH. DCFH-DA: Green fluorescence specific for ROS production. MERGE: Merged image of DCFH-DA fluorescence and bright field (DIC). Scale bar = 50 μm. (**B**) Relative fluorescence intensity of DCFH-DA staining. Note: Different lowercase letters in the bar chart indicate significant differences between groups (*p* < 0.05).

To investigate whether oxidative stress contributes to chilling injury and whether GSH confers antioxidant protection, intracellular ROS levels were assessed using the DCFH-DA probe. As shown in Figure 2A, fresh blastocysts displayed weak green fluorescence, indicating low basal ROS levels. In contrast, blastocysts preserved at 4 °C showed markedly increased fluorescence intensity, reflecting substantial ROS accumulation during hypothermic exposure. Quantitative analysis confirmed these observations, with the 4 °C group exhibiting significantly higher ROS levels (59.67 ± 1.76, n = 30) compared with the fresh control (42.67 ± 1.45, n = 30; *p* < 0.05). Importantly, supplementation with 4 mM GSH significantly reduced ROS production (46.00 ± 1.15, n = 30), restoring levels to values comparable to those of fresh embryos (*p* > 0.05). These results demonstrate that oxidative stress is a major contributor to chilling-induced embryonic injury, and GSH effectively maintains intracellular redox homeostasis by suppressing ROS accumulation during hypothermic storage.

### 3.3. GSH Reduces Apoptosis in Bovine Blastocysts Following 4 °C Preservation

**Figure 3:**
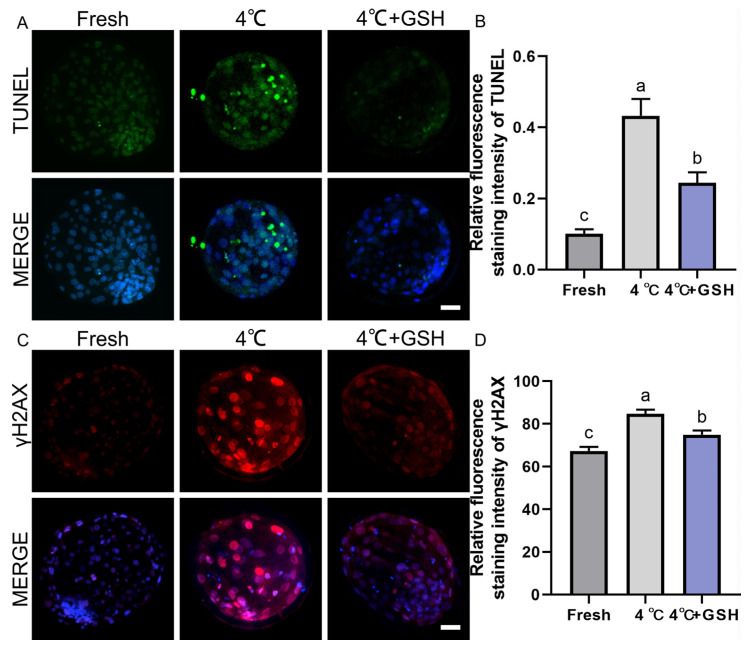
GSH protects nuclear integrity and inhibits apoptosis in 4 °C chilled bovine blastocysts; (**A**) Representative images of apoptosis detected by TUNEL staining. TUNEL: Green fluorescence labeling fragmented DNA, indicating apoptotic cells. MERGE: Merged result of TUNEL fluorescence and DAPI staining (blue, for all nuclei). Scale bar = 50 μm. (**B**) Relative fluorescence intensity of TUNEL staining (Apoptotic cell ratio). (**C**) Representative immunofluorescence images of γ-H2AX for DNA double-strand breaks. γ-H2AX: Red fluorescence indicating DNA damage. MERGE: Merged result of γ-H2AX fluorescence and DAPI staining (blue). Scale bar = 50 μm. (**D**) Relative fluorescence intensity of γ-H2AX staining. Note: Different lowercase letters in the bar charts indicate significant differences between groups (*p* < 0.05).

To assess chilling-induced apoptosis and determine whether GSH supplementation alleviates this damage, TUNEL staining was performed on fresh, 4 °C-preserved, and 4 °C + GSH blastocysts. As illustrated in Figure 3A, fresh embryos exhibited minimal TUNEL-positive nuclear signals, indicating low basal apoptotic activity. In contrast, blastocysts stored at 4 °C displayed a substantial increase in TUNEL-positive cells, reflecting DNA fragmentation and apoptosis activation after prolonged hypothermic exposure.

Quantitative analysis (Figure 3B) showed that the apoptotic cell ratio in the 4 °C group (0.43 ± 0.03, n = 30) was significantly higher than that of fresh embryos (0.10 ± 0.01, n = 30; *p* < 0.05). Supplementation with 4 mM GSH markedly reduced the apoptotic index to 0.24 ± 0.02 (n = 30), representing a significant improvement over the untreated 4 °C group (*p* < 0.05). Although apoptosis levels in the GSH-treated embryos remained slightly elevated compared with fresh controls (*p* < 0.05), the overall reduction indicates a strong protective effect. These results suggest that prolonged chilling activates apoptosis in bovine blastocysts, and GSH significantly mitigates apoptotic DNA fragmentation, partially restoring cellular integrity after hypothermic storage.

### 3.4. GSH Alleviates Chilling-Induced DNA Double-Strand Breaks

To further evaluate genomic stability, DNA double-strand breaks were assessed using γ-H2AX immunofluorescence. As shown in Figure 3C, fresh blastocysts exhibited weak γ-H2AX staining, consistent with low endogenous DNA damage. Following 4 °C preservation, embryos displayed a pronounced increase in γ-H2AX fluorescence, indicating extensive DNA damage due to chilling-induced oxidative stress. Quantitative results (Figure 3D) revealed that γ-H2AX fluorescence intensity in the 4 °C group (84.71 ± 1.15, n = 30) was significantly elevated compared with the fresh group (67.32 ± 1.13, n = 30; *p* < 0.05). Importantly, embryos preserved with 4 mM GSH showed substantially reduced γ-H2AX levels (74.98 ± 1.20, n = 30), which were significantly lower than those of the 4 °C group (*p* < 0.05). These findings indicate that chilling induces considerable DNA fragmentation in bovine blastocysts, and GSH supplementation effectively suppresses DNA double-strand break accumulation, likely through its potent antioxidant capacity and mitochondrial stabilization.

### 3.5. GSH Preserves Mitochondrial Activity in Bovine Blastocysts During 4 °C Preservation

**Figure 4:**
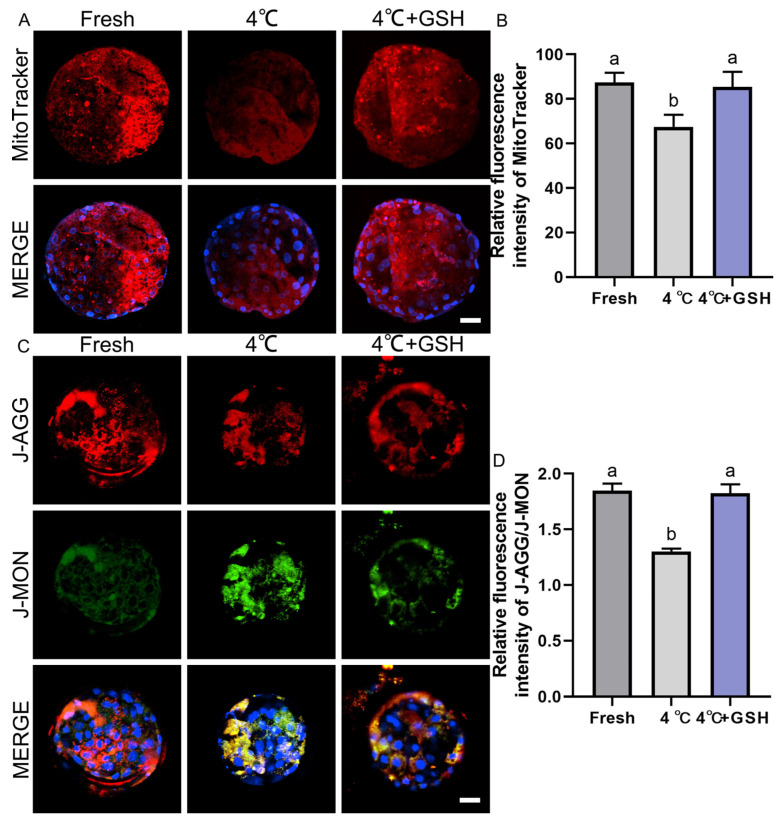
GSH preserves mitochondrial activity and membrane potential in 4 °C chilled bovine blastocysts; (**A**) Representative images of mitochondrial activity detected by MitoTracker Red probe. MitoTracker: Red fluorescence indicating active mitochondria. MERGE: Merged result of MitoTracker fluorescence and DAPI staining (blue). Scale bar = 50 μm. (**B**) Relative fluorescence intensity of MitoTracker staining (Mitochondrial activity). (**C**) Representative images of mitochondrial membrane potential (ΔΨm) detected by the JC-1 probe. J-AGG: Red fluorescence, indicating high ΔΨm (healthy mitochondria). J-MON: Green fluorescence, indicating low ΔΨm (damaged mitochondria). MERGE: Merged result of J-AGG, J-MON, and DAPI staining (blue). Scale bar = 50 μm. (**D**) Relative fluorescence ratio of J-AGG/J-MON (Mitochondrial membrane potential index). Note: Different lowercase letters in the bar charts indicate significant differences between groups (*p* < 0.05).

Mitochondria are highly sensitive to hypothermic stress, and impaired mitochondrial function is a major contributor to chilling-induced embryonic injury. To evaluate mitochondrial metabolic activity, MitoTracker Red staining was performed on fresh, 4 °C-preserved, and GSH-treated embryos. As shown in Figure 4A, fresh blastocysts exhibited strong red fluorescence uniformly distributed across blastomeres, indicative of robust mitochondrial activity. In contrast, blastocysts stored at 4 °C displayed visibly weakened MitoTracker fluorescence, suggesting suppressed mitochondrial function after prolonged chilling. Quantitative analysis (Figure 4B) confirmed a significant decline in mitochondrial activity in the 4 °C group (67.46 ± 3.11, n = 30) compared with fresh embryos (87.45 ± 2.39, n = 30; *p* < 0.05). Importantly, supplementation with 4 mM GSH significantly restored mitochondrial function (85.46 ± 3.85, n = 30), with activity levels not significantly different from the fresh group (*p* > 0.05). These results indicate that GSH effectively protects mitochondrial function during 4 °C storage, likely by alleviating oxidative stress and stabilizing mitochondrial ultrastructure.

### 3.6. GSH Restores Mitochondrial Membrane Potential Compromised by 4 °C Chilling

Mitochondrial membrane potential (ΔΨm) is an essential indicator of mitochondrial integrity and ATP-generating capacity. Using the JC-1 assay (Figure 4C), fresh blastocysts displayed strong red J-aggregate fluorescence, corresponding to high ΔΨm and healthy mitochondria. In contrast, the 4 °C group showed a marked shift from red to green fluorescence (J-monomers), indicating mitochondrial depolarization and functional impairment. Quantification of the J-aggregate/J-monomer fluorescence ratio (Figure 4D) revealed a significant decrease in ΔΨm in 4 °C-preserved embryos (1.30 ± 0.01, n = 30) compared with the fresh group (1.85 ± 0.04, n = 30; *p* < 0.05). This reduction demonstrates that hypothermic storage severely compromises mitochondrial polarization. Strikingly, embryos supplemented with 4 mM GSH during storage maintained a ΔΨm ratio of 1.83 ± 0.04 (n = 30), which was significantly higher than that of the 4 °C group (*p* < 0.05) and statistically indistinguishable from fresh embryos (*p* > 0.05). These results show that GSH effectively prevents mitochondrial depolarization and preserves mitochondrial bioenergetics during hypothermic preservation.

### 3.7. GSH Modulates the Expression of Antioxidant-, Apoptosis-, and Development-Related Genes in 4 °C-Preserved Bovine Blastocysts

**Figure 5:**
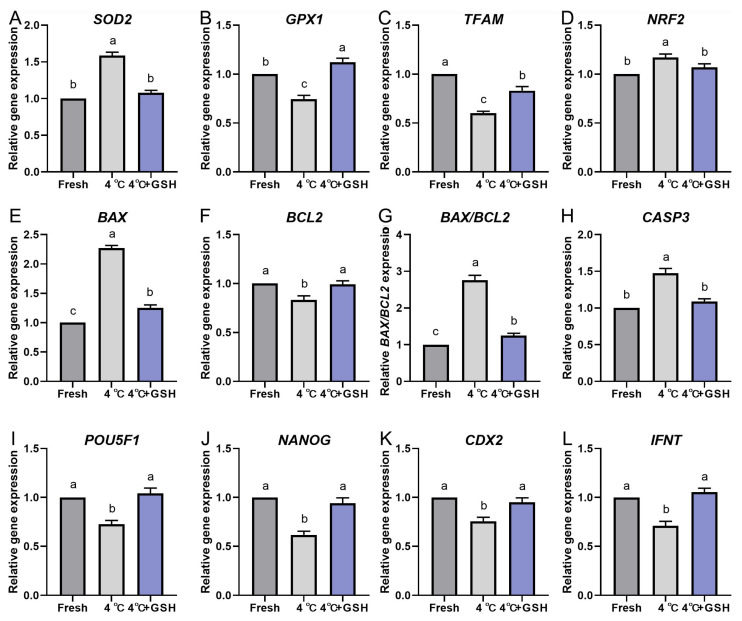
GSH modulates the relative mRNA expression of oxidative stress and apoptosis-related genes in 4 °C chilled bovine blastocysts; (**A**–**L**) Relative mRNA expression levels of key genes involved in antioxidant defense and apoptosis pathways. (**A**) Expression of SOD2 (Superoxide Dismutase 2). (**B**) Expression of GPX1 (Glutathione Peroxidase 1). (**C**) Expression of TFAM (Mitochondrial Transcription Factor A). (**D**) Expression of NRF2 (Nuclear Factor Erythroid 2-Related Factor 2). (**E**) Expression of the pro-apoptotic gene BAX. (**F**) Expression of the anti-apoptotic gene BCL-2. (**G**) Ratio of BAX/BCL-2 expression. (**H**) Expression of CASP3 (Caspase-3). (**I**) Expression of POU5F1 (POU Class 5 Homeobox 1). (**J**) Expression of NANOG (Nanog Homeobox). (**K**) Expression of CDX2 (Caudal-Type Homeobox 2). (**L**) Expression of IFNT (Interferon Tau). Note: Gene expression was quantified by qPCR and normalized to the internal control (β-actin). Data were calculated using the 2^−ΔΔCt^ method. Different lowercase letters indicate significant differences between the Fresh, 4 °C, and 4 °C + GSH groups (*p* < 0.05).

To further elucidate the molecular mechanisms underlying GSH-mediated protection during hypothermic storage, the expression profiles of key antioxidant, mitochondrial regulation, apoptosis, and embryonic developmental competence genes were evaluated via quantitative real-time PCR. The results are summarized in Figure 5A–L. Hypothermic storage at 4 °C significantly disrupted the antioxidant defense system of bovine blastocysts. Expression levels of SOD2, GPX1, TFAM, and NRF2 were all markedly downregulated in the 4 °C group compared with fresh controls (*p* < 0.05). These reductions indicate weakened mitochondrial resilience and decreased enzymatic ROS-scavenging capacity under chilling conditions.

Notably, supplementation with 4 mM GSH effectively reversed the decline in gene expression. Each of these antioxidant and mitochondrial regulatory genes was significantly upregulated in the GSH group compared with untreated 4 °C embryos (*p* < 0.05), reaching levels comparable to fresh controls. This suggests that GSH restores antioxidant transcriptional programs and supports mitochondrial homeostasis during low-temperature storage.

Chilling strongly activated apoptosis-related pathways. The expression of BAX and CASP3, two major pro-apoptotic markers, was significantly increased in the 4 °C group relative to fresh embryos (*p* < 0.05). Conversely, the anti-apoptotic gene BCL2 was markedly reduced, resulting in a significant elevation of the BAX/BCL2 ratio, an important indicator of apoptosis susceptibility. GSH supplementation significantly suppressed the chilling-induced increase in BAX and CASP3, while simultaneously elevating BCL2 expression (*p* < 0.05). Consequently, the BAX/BCL2 ratio was normalized to levels similar to fresh controls. These patterns align with the reduced apoptosis observed in TUNEL assays, demonstrating that GSH effectively inhibits the activation of apoptotic signaling cascades during 4 °C preservation.

To evaluate the effects of chilling and GSH on embryonic developmental potential, expression levels of key lineage and pluripotency-associated genes were quantified. Both POU5F1 and NANOG, critical regulators of inner cell mass (ICM) maintenance, were significantly downregulated following 4 °C preservation (*p* < 0.05). This suggests compromised pluripotency and impaired cellular quality of chilled embryos.

Similarly, IFNT, the principal maternal recognition signal produced by the trophectoderm (TE), was also significantly decreased after chilling, indicating reduced TE functional competence. Consistently, CDX2 expression—a key TE lineage marker—was likewise reduced following chilling, suggesting that TE identity is also transcriptionally sensitive to chilling stress, although to a lesser extent than ICM-associated markers.

Importantly, embryos preserved with GSH exhibited restored expression of POU5F1, NANOG, CDX2, and IFNT, with levels significantly higher than those in the untreated 4 °C group (*p* < 0.05) and comparable to fresh controls. These findings demonstrate that GSH supports embryonic cell fate stability and enhances developmental competence at the transcriptional level.

### 3.8. GSH Improves Cell Allocation and Developmental Potential of Bovine Blastocysts Following 4 °C Preservation

To assess whether GSH supplementation protects the structural integrity and developmental potential of bovine blastocysts during hypothermic storage, SOX2/CDX2 immunofluorescence staining was performed to examine inner cell mass (ICM) and trophectoderm (TE) lineage allocation. Total cell number and ICM/TE ratio were also quantified to evaluate embryonic quality.

**Figure 6:**
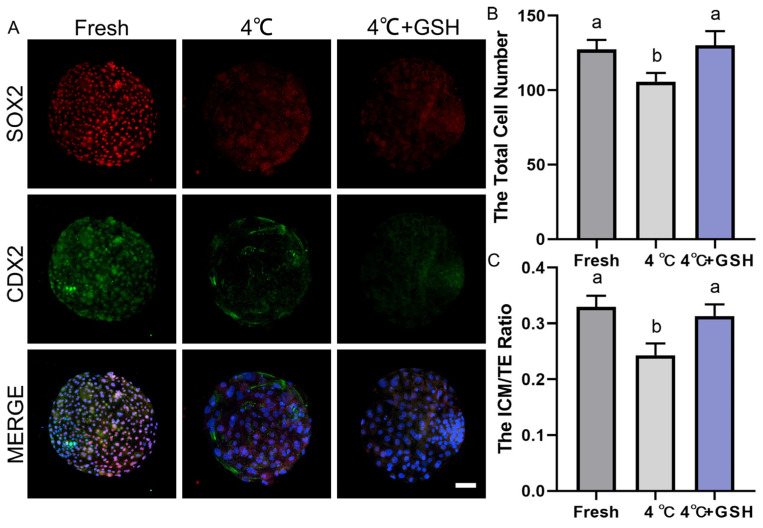
Effect of GSH on developmental potential and cell number in 4 °C-chilled bovine blastocysts; (**A**) Representative images of blastocyst developmental potential assessed by SOX2 and CDX2 co-staining. SOX2: Red fluorescence indicating the inner cell mass (ICM). CDX2: Green fluorescence indicating the trophectoderm (TE). MERGE: Overlay of SOX2 and CDX2 fluorescence with DAPI nuclear staining (blue). Scale bar = 50 μm. (**B**) Statistical diagram of the Total Cell Number in blastocysts after re-culture. (**C**) Statistical diagram of the ICM/TE ratio. Note: Different lowercase letters in the bar charts indicate significant differences between groups (*p* < 0.05).

As shown in Figure 6A, fresh blastocysts exhibited strong SOX2 (red) and CDX2 (green) signals, representing well-defined ICM and TE lineages, respectively. The spatial separation of SOX2^+^ ICM cells and CDX2^+^ TE cells reflects normal lineage specification. By contrast, blastocysts preserved at 4 °C showed clear deterioration in lineage structure, characterized by weakened SOX2 fluorescence and reduced CDX2 signal intensity. The boundaries between ICM and TE became less distinct, suggesting impaired cell fate stability and developmental competence. Remarkably, embryos preserved in 4 mM GSH displayed much stronger SOX2 and CDX2 expression compared with the untreated 4 °C group. The fluorescence patterns closely resembled those observed in fresh blastocysts, indicating that GSH effectively maintains ICM and TE lineage integrity during hypothermic storage.

Quantification of total cell numbers (Figure 6B) demonstrated that fresh blastocysts contained the highest number of cells. Hypothermic storage at 4 °C significantly reduced total cell number (*p* < 0.05), reflecting increased apoptosis, reduced proliferation, and general developmental suppression. However, supplementation with 4 mM GSH effectively restored total cell count to levels comparable with the fresh group (*p* > 0.05), and significantly higher than the untreated 4 °C group (*p* < 0.05). These findings suggest that GSH prevents chilling-induced cell loss and promotes post-warming recovery of embryonic proliferation potential.

As shown in Figure 6C, fresh embryos exhibited the highest ICM/TE ratio, reflecting a healthy balance between pluripotent ICM cells and differentiated TE cells. Chilling at 4 °C significantly decreased the ICM/TE ratio (*p* < 0.05), indicating that ICM cells are more vulnerable to cold-induced oxidative and apoptotic damage. Importantly, the addition of GSH significantly increased the ICM/TE ratio compared with the 4 °C group (*p* < 0.05), restoring the proportion of ICM cells to values similar to those of fresh blastocysts.

Together with the immunofluorescence results, these data demonstrate that GSH protects ICM cells from chilling-induced injury and preserves embryonic developmental competence.

## 4. Discussion

Short-term hypothermic preservation at 4 °C has emerged as an appealing alternative to conventional cryopreservation, yet its utility is limited by chilling-induced oxidative stress, mitochondrial dysfunction, and apoptosis [7,29]. Similar constraints have been reported in bovine, ovine, and porcine embryos, where low-temperature exposure consistently elevates ROS, disrupts mitochondrial membrane potential, and reduces developmental competence [7,12,14,30]. Compared with these earlier observations, the extent of oxidative imbalance and cellular injury in our chilled bovine blastocysts closely mirrors findings in ovine oocytes preserved at 5 °C and porcine morulae stored at 4–10 °C [12,18], highlighting the conserved susceptibility of mammalian embryos to hypothermic stress across species.

Antioxidant supplementation has been proposed as a strategy to improve low-temperature tolerance, but reported outcomes vary widely. For example, melatonin improved the survival of chilled ovine oocytes but showed limited benefit in bovine blastocysts [31,32], whereas α-tocopherol enhanced viability in porcine embryos but failed to correct mitochondrial dysfunction [33]. In contrast, GSH appears to provide broader protection: earlier studies showed that GSH supplementation improves spindle integrity in vitrified oocytes and reduces apoptosis in cryopreserved embryos [23,34]. The present work is consistent with these findings, suggesting that GSH exerts a more comprehensive effect than many other antioxidants—likely due to its dual role as a direct ROS scavenger and as a regulator of NRF2-mediated antioxidant gene expression. Compared with compounds that act through a single pathway (e.g., SOD mimetics), GSH’s ability to enhance endogenous SOD2, GPX1, and TFAM expression may explain its comparatively stronger impact on restoring mitochondrial homeostasis.

The protective mechanism of exogenous GSH likely involves both extracellular and intracellular pathways. GSH can act directly as an extracellular antioxidant to scavenge ROS in the preservation medium. Additionally, through the γ-glutamyl cycle, extracellular GSH is hydrolyzed by γ-glutamyl transpeptidase (γ-GT) into its constituent amino acids (glutamate, cysteine, and glycine), which are then transported into the blastomeres to fuel intracellular GSH synthesis [35]. This replenishes the intracellular antioxidant pool, stabilizing mitochondrial function and preventing oxidative DNA damage.

It is noteworthy that while 4 mM GSH significantly improved survival and hatching rates compared to the control and 2 mM groups, increasing the concentration to 8 mM did not yield further benefits. This plateau suggests a mechanism of intracellular saturation or the ‘antioxidant paradox’ [36]. Recent studies indicate that while high levels of ROS are detrimental, physiological levels of ROS are indispensable acting as secondary messengers for essential developmental events, such as cell cycle progression and compaction [37,38]. Consequently, excessive antioxidant supplementation (8 mM) might induce ‘reductive stress,’ suppressing these vital physiological signals and counteracting the benefits of ROS scavenging [38]. Therefore, 4 mM GSH appears to be the optimal concentration that mitigates oxidative injury without compromising the redox signaling required for bovine blastocyst homeostasis. Lineage integrity also represents a useful metric for evaluating preservation methods. Previous chilling studies reported substantial reductions in ICM cell number and altered SOX2/CDX2 localization, particularly in species with high metabolic sensitivity such as bovine and buffalo embryos [39,40]. Our GSH-supplemented embryos maintained lineage allocation patterns more closely resembling those reported in optimized vitrification systems rather than untreated chilled embryos, suggesting that GSH narrows the quality gap between hypothermic preservation and cryopreservation—though it does not yet fully eliminate it. This distinction is important: while vitrification remains superior for long-term storage, improved hypothermic strategies may be sufficient for short-term transport and synchronization tasks, provided that embryonic quality is not significantly compromised.

From an applied perspective, hypothermic storage has been explored for decades, yet adoption in cattle production has been limited by inconsistent survival outcomes [7,30]. Previous attempts using commercial holding media or metabolic inhibitors extended preservation to only 2–3 days with acceptable viability [41,42]. In comparison, the seven-day storage window achieved here with GSH supplementation exceeds the typical preservation limits reported for bovine embryos and approaches the performance observed in rabbit and murine models, where embryos naturally exhibit higher cold tolerance. This suggests that GSH partially compensates for the intrinsic cold sensitivity of bovine embryos, offering a practical extension of transport time without requiring cryogenic infrastructure.

Despite these advances, further work is necessary to clarify whether the developmental improvements observed in vitro translate into higher pregnancy rates after transfer—an area largely unaddressed in the hypothermic preservation literature. Comparative studies evaluating GSH alongside other antioxidants (e.g., cysteamine, resveratrol, CoQ10) or metabolic regulators (e.g., AMPK modulators) will help determine whether combinational approaches provide additive or synergistic benefits. Additionally, since epigenetic drift has been reported in chilled embryos of several species [43,44,45], it will be important to assess whether GSH helps maintain methylation stability and imprinting integrity during extended low-temperature storage.

Collectively, these findings demonstrate that exogenous GSH protects bovine blastocysts from chilling injury by suppressing ROS accumulation and stabilizing mitochondrial function. However, a limitation of the present study is the lack of in vivo transfer data. While in vitro quality indicators (survival, cell number, gene expression) were significantly improved, future studies involving embryo transfer experiments are necessary to confirm whether these cellular improvements translate into higher pregnancy and calving rates.

Overall, by situating our findings within the context of prior preservation strategies, it becomes clear that GSH offers a more robust and mechanistically comprehensive means of enhancing embryo resilience during hypothermic storage. While not a replacement for cryopreservation, GSH-supported hypothermic preservation represents a promising, pragmatic approach for short-term embryo handling, transportation, and genetic resource management in the cattle industry.

## 5. Conclusions

This study clearly demonstrates the protective role of exogenous GSH during the short-term chilling (4 °C) of bovine blastocysts. The optimal dose of 4 mM GSH significantly improved the post-chilling survival and hatching rates. Mechanistically, we show that GSH acts by scavenging excessive ROS and concurrently restoring the chilling-induced decline in mitochondrial function (ΔΨm and activity). This dual action effectively inhibited cellular apoptosis and DNA damage, as evidenced by reduced γ-H2AX and Caspase-3 activity. Consequently, GSH-treated blastocysts maintained higher cell numbers, a better ICM/TE ratio, and enhanced expression of key developmental genes (e.g., POU5F1 and NANOG). In summary, GSH represents a safe and effective strategy to mitigate chilling injury by preserving mitochondrial integrity and suppressing apoptosis, thus significantly enhancing the viability and developmental competence of bovine embryos stored under hypothermic conditions.

## Data Availability

We declared that materials described in the manuscript, including all relevant raw data, will be freely available to any scientist wishing to use them for non-commercial purposes, without breaching participant confidentiality.
